# Lesions of the posterior paraventricular nucleus of the thalamus attenuate fear expression

**DOI:** 10.3389/fnbeh.2014.00094

**Published:** 2014-03-20

**Authors:** Yonghui Li, Xinwen Dong, Sa Li, Gilbert J. Kirouac

**Affiliations:** ^1^Key Laboratory of Mental Health, Institute of Psychology, Chinese Academy of SciencesBeijing, China; ^2^Department of Oral Biology, Faculty of Dentistry, University of ManitobaWinnipeg, MB, Canada; ^3^Department of Psychiatry, Faculty of Medicine, University of ManitobaWinnipeg, MB, Canada

**Keywords:** paraventricular nucleus, fear, learning, midline thalamus, motivation

## Abstract

The paraventricular nucleus of the thalamus (PVT) has generated interest because of its strong projections to areas of the brain associated with the regulation of emotional behaviors. The posterior aspect of the PVT (pPVT) is notable for its projection to the central nucleus of the amygdala which is essential for the expression of a conditioned fear response. The present study was done to determine if the pPVT is involved in the expression of fear by examining the effect of post-conditioning lesions of the pPVT. Male rats were trained to bar press for food pellets on a variable ratio schedule. Fear conditioning was done using auditory tones (30 s) that co-terminate with footschocks (0.65 mA, 1.0 s). Rats were anesthetized 24 h later and small bilateral electrolytic lesions of the pPVT were made. Fear expression to the tone was assessed using suppression of bar-pressing and freezing after one week of recovery from the surgical procedure. Small bilateral lesions of the pPVT increased bar-pressing for food and decreased freezing during the presentation of the conditioned tone. Lesions of the pPVT had no effect on fear extinction, fear conditioning to a novel tone, or the motivation for food as assessed using a progressive ratio (PR) schedule. The results of the experiment support a role for the pPVT in fear expression. In contrast, the pPVT does not appear to be involved in fear learning or extinction nor does it appear to play a role in the motivation of rats to bar press for food.

## Introduction

Classical (Pavlovian) fear conditioning refers to a form of learning that occurs when a neutral stimulus such as a tone (conditioned stimulus, CS) starts to acquire fear properties through repeated temporal pairings with aversive events like an electrical shock (unconditioned stimulus, US; LeDoux, 1993, 2000). As the CS-US relationship is learned, the behavioral and physiological responses of fear (e.g., freezing in rodents) occur in the presence of the tone alone.

Several decades of research has revealed the essential components of the neural circuitry required for the expression of conditioned fear (LeDoux, 1993, 2000; Ehrlich et al., 2009; Pape and Pare, 2010). The fear system is composed of a projection from the medial part of the central nucleus of the amygdala (CeA_M_; LeDoux, 1993, 2000; Ehrlich et al., 2009; Pape and Pare, 2010) to areas of the hypothalamus and brainstem that regulate the expression of the behaviors and autonomic responses associated with fear (Krettek and Price, 1978a; Veening et al., 1984; LeDoux et al., 1988; Petrovich and Swanson, 1997; Dong et al., 2001). Input to the central nucleus of the amygdala comes from the basolateral amygdala which stores associations between cues and aversive stimuli (Blair et al., 2001; Ehrlich et al., 2009; Josselyn, 2010; Maren, 2011). The modulation of conditioned fear is complex with neural signals traveling from the basolateral amygdala to the central nucleus of the amygdala through a network of inhibitory neurons (Krettek and Price, 1978b; Paré and Smith, 1993, 1998; Paré et al., 1995). In addition, the prelimbic and infralimbic portions of the medial prefrontal cortex modulate fear expression by way of interconnections between these areas of the cortex and the amygdala (Quirk and Mueller, 2008; Maren, 2011).

The anatomical connections between the dorsal midline thalamus and other regions of the brain involved in conditioned fear suggest that this part of the thalamus could also modulate fear. For instance, both the mediodorsal nucleus and the paraventricular nucleus of the thalamus (PVT) are interconnected with prelimbic and infralimbic cortices, which regulate fear expression and extinction, respectively (Vertes, 2004; Vertes and Hoover, 2008; Li and Kirouac, 2012). In addition, the PVT is in a unique position to influence fear through direct projections to the central nucleus of the amygdala and the basolateral amygdala (Li and Kirouac, 2008; Vertes and Hoover, 2008). Indeed, some studies found support for a role for the dorsal midline thalamus in the regulation of conditioned fear (Li et al., 2004; Padilla-Coreano et al., 2012). For instance, lesions of the dorsal midline thalamus impaired the acquisition and expression of freezing to a conditioning context (Li et al., 2004). In another study, temporary inactivation of neurons in the midline thalamus using microinjections of a GABA agonist prevented freezing to a tone previously associated with footshocks (Padilla-Coreano et al., 2012). However, it is impossible to identify the specific nuclei that are responsible for these effects since a relatively large area of the thalamus was affected by these experimental manipulations.

The PVT represents a viable candidate for modulating conditioned fear because of its projections to the prefrontal cortex and the amygdala. Reports of an increase in cFos in the PVT when rats were placed in the context in which footshocks were previously given also support a role for the PVT in learned fear (Beck and Fibiger, 1995; Yasoshima et al., 2007). In addition, the observation that microinjections of muscimol in the midline thalamus increases cFos expression in the lateral part of the central nucleus of the amygdala (CeA_L_), which has been shown to receive direct projections from the PVT (Li and Kirouac, 2008; Vertes and Hoover, 2008), points to a potential mechanism for PVT to modulate fear (Padilla-Coreano et al., 2012). The present study was done to examine the effect of making small bilateral lesions of the posterior portion of the PVT (pPVT) to determine if this area of the midline thalamus is involved in fear. This region of the PVT was targeted because of its dense projection to the CeA_L_ (Li and Kirouac, 2008).

## Materials and methods

### Animals and housing

Male Sprague-Dawley rats (Charles River, Beijing) weighing 260–280 g at the time of arrival at the laboratory were housed individually in cages kept in a room maintained on a 12 h/12 h light/dark cycle (lights on at 07:00) with controlled temperature (20–24°C) and humidity (40–70%). All the rats had free access to food and water and were handled for 2 min on alternate days during a 7-day adaptation period. The rats were then restricted to 10–15 g of food per day until they reached 85% of their free-feeding weights. During the food restriction period, rats were acclimated to 45 mg sugar pellets (Bio-Serv, Frenchtown, NJ, USA) which were used for subsequent bar-pressing training. All the behavioral training and tests were done in the light cycle of the day (09:00–17:00). The experimental procedures conform to the National Institutes of Health Guide for Care and Use of Laboratory Animals (Publication No. 85-23, revised 1985) and the experimental protocol was approved by Research Ethics Review Board of Institute of Psychology, Chinese Academy of Sciences.

### Bar-pressing training

Rats were trained to bar press for food using a standard operant conditioning chamber (MED Associates, St. Albans, VT, USA). The chamber consisted of a stainless steel grid floor for the delivery of footshocks, a lever on one wall for bar-pressing, and a speaker mounted to the ceiling of the chamber for the delivery of tones. The chamber was housed in a sound-attenuating box to reduce ambient noise to 60 dB. Pellet delivery was controlled by a computer running commercially available software (MED associates, St. Albans, VT, USA). The rats were trained to bar press for sugar pellets during 30-min sessions using a variable interval (VI) schedule. The initial reinforcement ratio was VI2 (rats received one pellet for each bar press after 2 s had elapsed) and the ratio was increased gradually (VI5, VI10, VI30, VI60) until rats reached a minimum of 15 presses per minute (7–10 days of training).

### Fear conditioning

The CS was a 30 s tone (4 kHz at 80 dB) and the US was a scrambled footshock (0.65 mA, 1.0 s) that co-terminated with the tone. Rats were exposed to five habituation trials of the tone alone followed by ten conditioning trials (tones paired with shocks, intertrial interval (ITI) = 60–180 s). The presence of a conditioned fear response was assessed using percent freezing time and suppression of bar-pressing. The activity of the rats was recorded using a digital camera suspended in the ceiling of the chamber. The amount of freezing during the tone presentation was quantified by commercially available software (Ethovision, Noldus, Wageningen, Netherland) with freezing defined as the complete lack of movement except for those related to breathing. Percent freezing time was expressed as the amount of time spent freezing during the tone/30 s × 100. The number of bar presses during the tone and 30 s prior to the tone (pre-CS) was used to calculate the conditioned suppression ratio which was expressed as responses during the CS/responses during the pre-CS + responses during the CS. Consequently, a suppression ratio of 0.5 is an indication of a lack of fear whereas a suppression ratio of 0 is indicative of intense fear.

### Lesions

Lesions of the midline thalamus were done 24 h after fear conditioning. Rats were anesthetized with equithesin (0.3 ml/100 g, i.p.) and placed in a Stoelting stereotaxic frame (Stoelting Co. Wood Dale, IL, USA). Bilateral electrolytic lesions of the pPVT were done using bipolar stainless-steel electrodes (RH SNE-100 × 50 mm, David Kopf Instruments, CA, USA) that were lowered in the brain at a 10° angle from the midline on one side of the sagittal sinus. The coordinates were 3.1 mm posterior to bregma, 0.7 mm and 1.2 mm lateral to the midline (for contralateral and ipsilateral lesions, respectively), and 5.3 mm ventral to the skull (incisor bar at 3.3 mm below intraaural line). The lesions were made using anodal constant direct current (25 µA, 120 s). The sham lesion animals were exposed to the same procedure except that no electrical current was passed through the electrode. The incision was sutured and the rats were given penicillin (80,000 units, i.m.). The rats were allowed 7 days of recovery before examining the effects of the lesions on conditioned fear.

### Behavioral tests

The ability of rats to bar press was examined at one week after the lesions were made. One or two bar-pressing training sessions were done to recover the bar-pressing response in rats that performed below the baseline criterion of 15 presses per min. Fear expression and extinction were assessed the next day by presenting 15 conditioning tones to the rats placed in the conditioning chamber. Locomotion and freezing were assessed in the periods before the first tone presentation (pre-CS, 180 s) to exclude the non-specific effect of lesion on movement. The recall of the extinction memory was examined the next day by presenting the tones five more times. Two days later, the rats were placed in a novel plastic chamber with black walls and white lid (MacroAmbition S and T Development Co., Ltd., Beijing, China) in a different room for fear conditioning to a new tone. In this case, the CS was a 2 kHz pure tone lasting 30 s (80 dB) that co-terminated with footshocks (1.0 mA, 1.0 s). The rats were placed into the chamber and allowed to explore the novel context for 2 min before receiving three CS-US pairings (ITI = 60–180 s). Freezing duration during the tone presentation was quantified as described above.

Two days after the second fear conditioning procedure, experiments were done to determine if pPVT lesion interfered with the motivation of rats to bar press for food when they were exposed to a progressive ratio (PR) procedure. The response requirement for getting a food pellet was incremental (1, 2, 4, 6, 9, 12, 15, 20, 25, 32, 40, 50, 62, 77, 95, 118, 145, 178, 219) as derived from a PR equation (Richardson and Roberts, 1996). The PR session lasted 60 min with the last rewarded bar press defined as the breakpoint. The rats were food deprived for 24 h and were re-exposed to the same PR schedule to evaluate their motivation for food in a food deprived state.

### Lesion verification

Rats were anesthetized with chloral hydrate (40 mg/kg) and perfused with heparinized saline followed by 4.0% paraformaldehyde in 0.1 M phosphate buffer (PB; pH 7.4). Coronal sections of the posterior thalamus were obtained at 100 µm using a vibratome which were subsequently stained for Nissl substance using Cresyl Violet. The lesions were mapped to identify rats with lesions that were restricted to the pPVT and those with lesions that involved the pPVT as well as parts of the mediodorsal and intermediodorsal nuclei.

### Data collection and analysis

Data for percent freezing and the suppression ratio were averaged over three tone presentations (five test blocks). The data for each test block was used for all subsequent statistical tests involving fear expression (first test block) and extinction (all five test blocks). Data from the first test block (first three trials) were used for statistical analysis of the extinction test. The breakpoint (highest PR reached) was calculated for each animal and used to assess motivation for food. Statistical analysis was done using two-way and one-way ANOVA as appropriate (with repeated measure for the extinction data). *Post-hoc* LSD tests were used to compare group differences. A value of *p* < 0.05 was considered to be significant and the data were presented as mean ± SEM.

## Results

**Figure 1:**
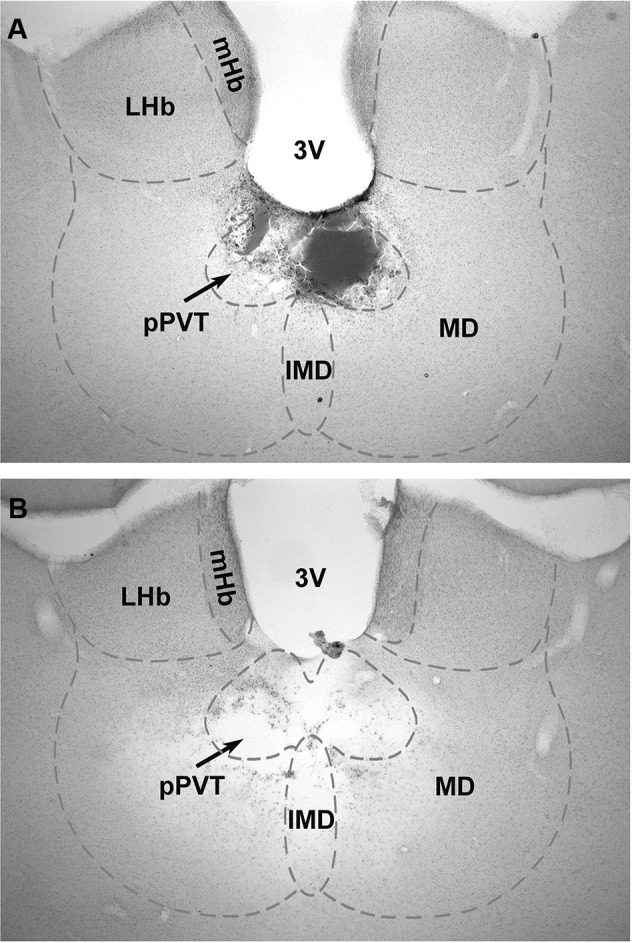
**Examples of lesions of the pPVT**. The location of the lesions as verified from histological sections of the brain were used to group the data into pPVT lesion (complete lesions limited to the pPVT); **(A)** and midline lesion (complete lesion of the pPVT and surrounding area including the mediodorsal and intermediodorsal nuclei); **(B)** groups. 3V, third ventricle; IMD, intermediodorsal nucleus; LHb, lateral habenular; mHb, medial habenular; MD, mediodorsal nucleus; pPVT, posterior paraventricular nucleus of the thalamus.

**Figure 2:**
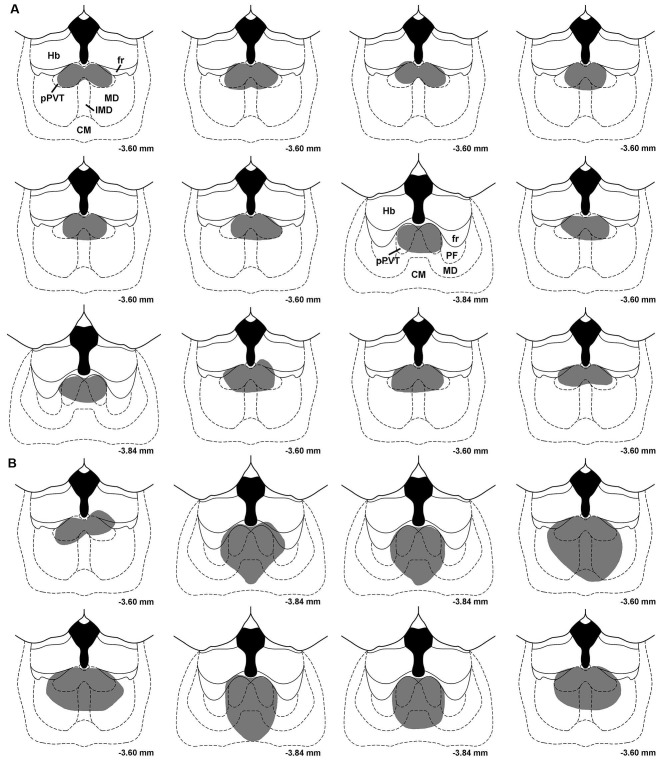
**Drawings showing the location of midline thalamic lesions**. Each section represents subjects that were assigned to the pPVT lesion group **(A)** and the midline lesion group **(B)**. CM, centromedial nucleus; fr, fasciculus retroflexus; Hb, habenula; IMD, intermediodorsal nucleus; MD, mediodorsal nucleus; PF, parafascicular nucleus; pPVT, posterior paraventricular nucleus of the thalamus. Numbers represent the distance from bregma.

Figure 1 shows a typical lesion largely restricted to the pPVT (Figure 1A) and a larger lesion involving more of the midline thalamus (pPVT and surrounding area; Figure 1B). Subjects were assigned to the pPVT lesion group (pPVT only;* n* = 13; Figure 2A), midline lesion group (pPVT and parts of the mediodorsal and intermediodorsal nuclei; *n* = 7; Figure 2B), non-pPVT lesion (no visible lesion because the electrode was placed in the third ventricle or small lesions that were dorsolateral to the pPVT in the medial habenula; *n* = 12) and sham lesion group (*n* = 5) based on the location of the lesions. Data from the sham group and non-pPVT lesion group were pooled together (control lesion group; *n* = 17) since both groups had similar fear levels and extinction rates as assessed by the suppression ratio [*F*_(4,60)_ = 1.049,* p* = 0.39, for interaction effect; and *F*_(1,15)_ = 0.799,* p* = 0.386, for group differences] and freezing [*F*_(4,60)_ = 0.678, *p* = 0.61, for interaction effect; *F*_(1,15)_ = 0.033, *p* = 0.857, for group differences] expressed over the 15 tone presentations of the expression/extinction phase of the tests.

**Figure 3:**
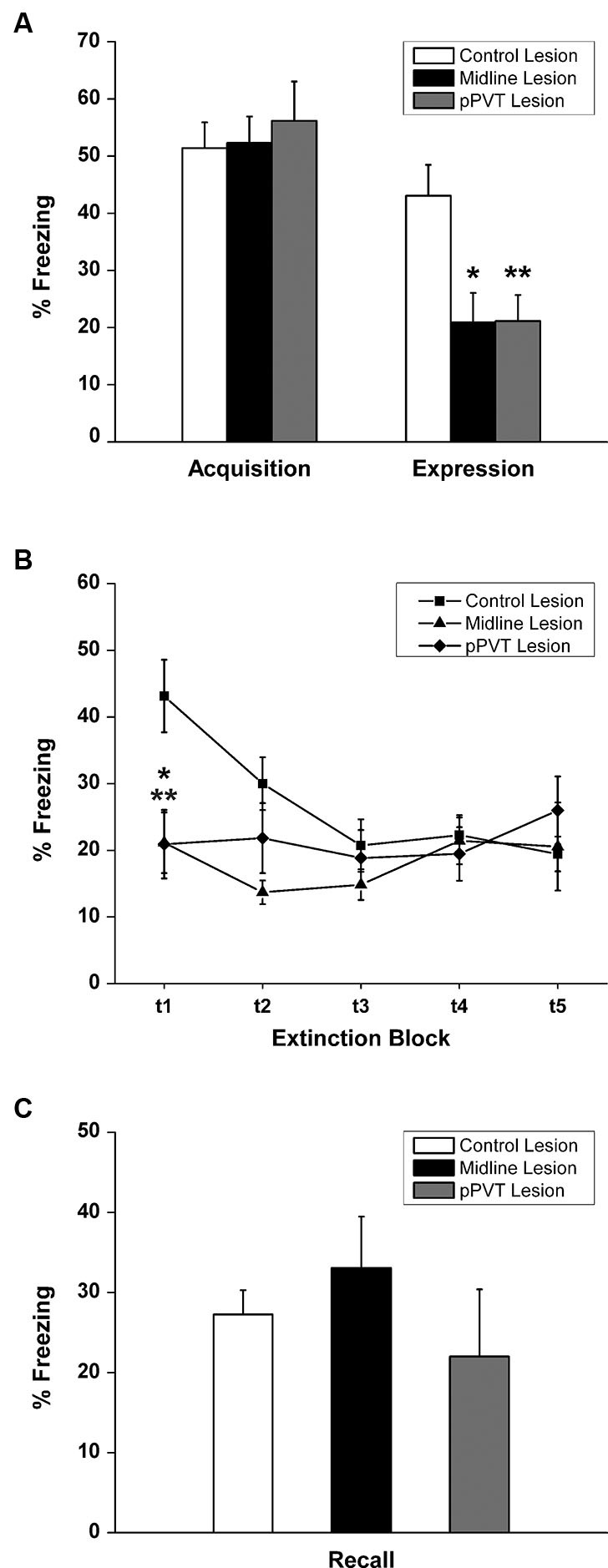
**Effect of pPVT lesions on freezing to fear-conditioning tones. (A)** Different experimental groups displayed similar amounts of freezing prior to electrolytic lesions of the pPVT. In contrast, freezing was attenuated in groups of rats that had pPVT and midline lesions when they were compared to the control lesion group. Data points are represented as percent freezing over the first three tone presentations (first test block). **(B)** The different groups showed similar extinction of freezing behavior over subsequent presentations of the tone. Each data point represents percent freezing for three consecutive tone presentations (one test block) with the first data point representing the same data as in Figure 2A. **(C)** Lesions of the pPVT or the midline thalamus had no effect on recall of the extinguished fear memory as measure by percent freezing over three tone presentations. *p < 0.05; **p < 0.01. Results are presented as mean ± SEM.

**Figure 4:**
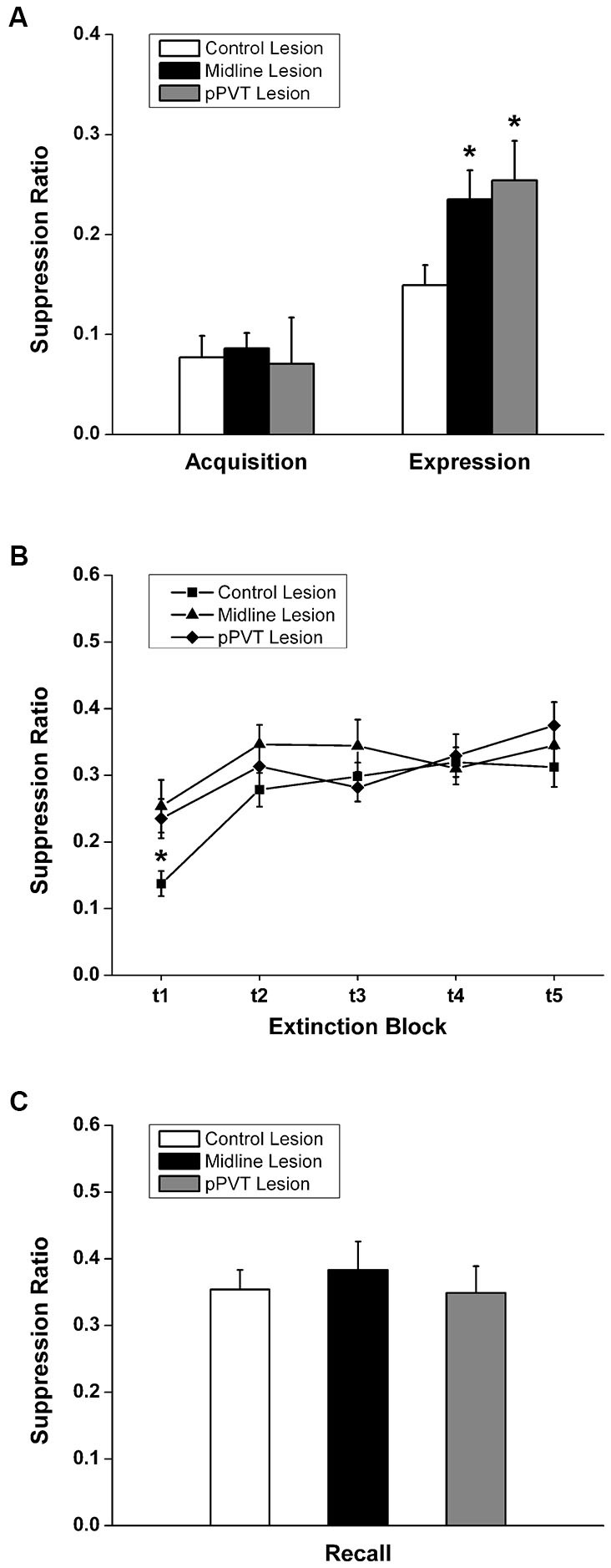
**Effect of pPVT lesions on suppression of bar-pressing to fear-conditioning tones. (A)** Different experimental groups displayed similar amounts of bar-pressing prior to electrolytic lesions of the pPVT. In contrast, bar-pressing was attenuated in groups of rats that had pPVT and midline lesions when they were compared to the control lesion group. Data points are represented as the suppression ratio over the first 3 tone presentations (first test block). **(B)** The different groups showed similar extinction of bar-pressing over subsequent presentations of the tone. Each data point represents the suppression ratio for three consecutive tone presentations (first test block) with the first data point representing the same data as in Figure 3A. **(C)** Lesions of the pPVT or the midline thalamus had no effect on recall of the extinguished fear memory as measured by the suppression ratio over three tone presentations. *p < 0.05. Results are presented as mean ± SEM.

**Figure 5:**
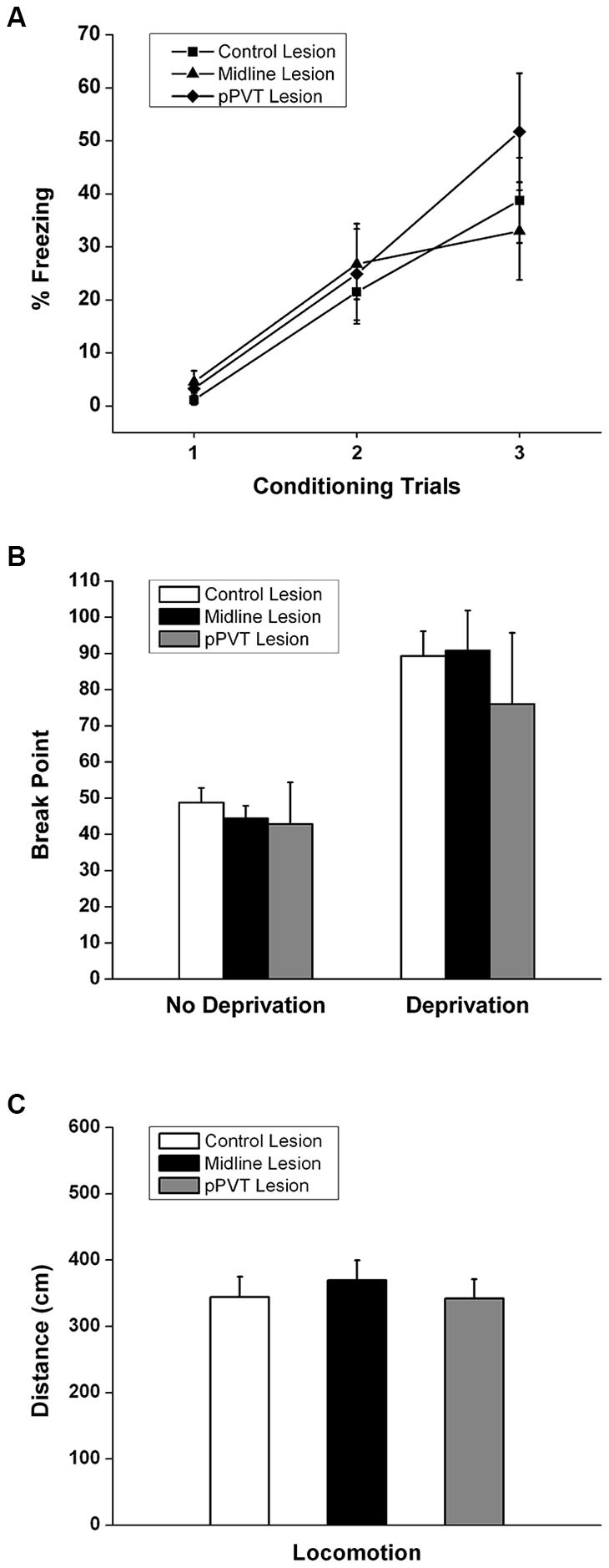
**Effect of pPVT lesions on the acquisition of a novel fear, motivation for food reward, and locomotor activity. (A)** Lesions of the pPVT or the midline thalamus had no effect on the freezing displayed in rats exposed to a new fear conditioning protocol. Each data point represents a single conditioning trial. **(B)** Lesions of the pPVT or the midline thalamus did not affect the breakpoint in food deprived or non-deprived rats. **(C)** Lesions of the pPVT did not affect the amount of locomotor activity displayed by rats during the pre-CS period of expression test. Results are presented as mean ± SEM.

A similar amount of fear to the tone as measured by freezing [*F*_(2,34)_ = 0.182, *p* = 0.835; Figure 3A] or the suppression ratio [*F*_(2,34)_ = 0.097, *p* = 0.908; Figure 4A] was displayed by the pPVT, midline and control lesion groups during fear conditioning (before the lesion surgery). There was a significant group difference in freezing during the fear expression test [*F*_(2,34)_ = 5.83, *p* = 0.007; Figure 3A]. Compared to the control lesion group, the pPVT lesion group [*p* = 0.004] and the midline lesion group [*p* = 0.018] showed significant decreases in freezing. There was also a significant difference between groups on the suppression ratio during the expression test [*F*_(2,34)_ = 4.215, *p* = 0.023; Figure 4A]. Compared to the control lesion group, the pPVT lesion group [*p* = 0.022] and the midline lesion group [*p* = 0.023] showed significant decreases in the suppression ratio. The one-way ANOVA indicated no differences in locomotion [*F*_(2,34)_ = 0.161, *p* = 0.852; Figure 5C] and freezing [*F*_(2,34)_ = 0.198,* p* = 0.821, data not shown] between the different groups.

When considering the groups together, the two-way ANOVA with repeated measures revealed that the fear memory was significantly extinguished using the suppression ratio [*F*_(4,136)_ = 10.35, *p* < 0.001 for the main effect for test block] and freezing [*F*_(4,136)_ = 4.41, *p* = 0.002 for the main effect for test block] as measures of fear. Furthermore, there was a significant interaction effect between the test blocks and lesions on freezing [*F*_(8,136)_ = 4.99, *p* < 0.001] and simple effect analysis was done to confirm that only the control lesion group showed a significant extinction effect [*F*_(4,136)_ = 15.81, *p* < 0.001; Figure 3B]. The pPVT and midline lesion groups showed no extinction using freezing as a measure of extinction because fear expression was already inhibited to extinction levels in the first test block. In contrast, lesions had no effect on the extinction of fear memory as measured by suppression ratio as specified by the two-way ANOVA analysis showing a lack of a main effect for lesions [*F*_(2,34)_ = 1.89,* p* = 0.167; Figure 4B] or an interaction effect between lesions and test blocks [*F*_(8,136)_ = 1.55, *p* = 0.144; Figure 4B]. Finally, the one-way ANOVA reveled no difference between lesion groups in recall test for extinction memory as assessed by freezing [*F*_(2,34)_ = 0.331,* p* = 0.720; Figure 3C] or suppression ratio [*F*_(2,34)_ = 0.193,* p* = 0.826; Figure 4C].

The effect of lesions of the pPVT on the acquisition of novel fear was also examined. Rats acquired a novel fear within three trials [*F*_(2,68)_ = 36.525, *p* < 0.001; Figure 5A]. However, pPVT or midline lesions had no effect on fear acquisition [*F*_(4,68)_ = 1.015, *p* = 0.406 for the interaction between lesion and trial; *F*_(2,34)_ = 0.297,* p* = 0.745 for group differences; Figure 5A]. Finally, neither the pPVT lesions or the midline lesions had any effect on the motivation to bar press in non-deprived or in food deprivation rats [*F*_(2,34)_ = 0.276, *p* = 0.360 for the interaction between lesion and deprivation state; *F*_(2,34)_ = 0.446,* p* = 0.646 for group differences; Figure 5B].

## Discussion

The results of the present study support the view that the pPVT plays a role in learned fear. This is indicated by the observation that small lesions restricted to the pPVT attenuated fear expression to the same extent as larger lesions of the pPVT and portions of other midline thalamic nuclei. In contrast, lesions of the pPVT had no effect on the extinction rate of the fear memory nor did it interfere with the acquisition of fear to a novel tone. In addition, the observation that the motivation for rats to bar press for food was unaffected by lesions of the pPVT indicates that this part of the PVT may preferentially regulate negative emotional behavior.

A number of studies have provided evidence for a role for the dorsal midline thalamus in fear (Li et al., 2004; Lee et al., 2012; Padilla-Coreano et al., 2012). In one study, large lesions of the mediodorsal nucleus and other midline thalamic nuclei made before and after the exposure of rats to footshocks were reported to interfere with fear expression to the shock context (Li et al., 2004). In a second study, microinjections of a GABA agonist in the dorsal midline thalamus prevented fear to a tone previously associated with footshocks but had no effect on the acquisition or the extinction of the fear (Padilla-Coreano et al., 2012). A third study was able to show that tonic activity of neurons in the mediodorsal nucleus was correlated with the level of fear extinction in conditioned mice, indicating a specific role for the mediodorsal nucleus in fear extinction (Lee et al., 2012). Except for the latter study, the other studies were not designed to address the specific location of the neurons in the midline thalamus that were involved in modulating fear.

The pPVT represents a possible candidate for the fear attenuating effects of inactivation of the dorsal midline thalamus because of the direct projections between the pPVT and the amygdala (Li and Kirouac, 2008; Vertes and Hoover, 2008). Indeed, other dorsal midline thalamic nuclei do not project directly to the basolateral or the central nucleus of the amygdala (Li and Kirouac, 2008). The present study was done to evaluate the role of the pPVT in learned fear by examining the effects of removing the influence of neurons located only in the pPVT in rats that had been subjected to an auditory fear conditioning protocol. The choice of electrolytic lesions for these experiments was based on the fact that it provides a means of making relatively small focal damage to an area of the brain that can be precisely verified in histological sections. Consistent with a role for the pPVT in fear, we found that bilateral lesions of the pPVT reduced fear as demonstrated by a reduction in freezing and a decrease in suppression of bar-pressing. We were unable to determine if lesions had an effect on extinction using freezing as a fear response because this measure was very low in the lesion groups. However, we were able to show using suppression of bar-pressing as a measure of fear that extinction and recall of the fear memory remained unaffected in rats with pPVT lesions. These results are in line with recent observations that injections of the GABA agonist muscimol in the dorsal midline thalamus attenuated fear but had no effect on extinction or recall of the extinction memory (Padilla-Coreano et al., 2012). The result of our experiments also shows that pPVT lesions produced similar effects as larger lesions involving pPVT, intermediodorsal and mediodorsal nuclei. This indicates that neurons located in the pPVT were contributing to the conditioned fear responses observed in the present study. It is also apparent that the motivation for rats to bar press for food was not compromised in rats with pPVT lesions indicating specificity in the effect of the lesions on fear. Moreover, freezing to a novel conditioned fear was not impaired nor was the level of locomotor activity displayed in the pre-CS period in rats with lesions of pPVT indicating that these rats did not suffer from any motor or performance deficits.

A possible mechanism by which the pPVT could regulate fear was provided by an experiment in which cFos was used to identify neurons that may have inhibited freezing in conditioned rats following inactivation of the dorsal thalamus (Padilla-Coreano et al., 2012). An increase in cFos was observed in the CeA_L_ whereas a decrease was noted for the CeA_M_ in rats that had received muscimol (low fear state) compared to those that received saline (high fear state). These results suggested that in fear conditioned rats, neurons in the region of the dorsal midline thalamus exert inhibitory influence on a population of CeA_L_ neurons that in turn inhibit neurons in the CeA_M_ (Padilla-Coreano et al., 2012). This mechanism is consistent with a proposed model in which a subpopulation of neurons in the CeA_L_ exerts a tonic inhibitory influence on fear expressing projection neurons in the CeA_M_ (Wilensky et al., 2006; Ehrlich et al., 2009; Pape and Pare, 2010). This suggests that fear-attenuating effects of inactivation of the pPVT would likely be due to the removal of an inhibitory influence on the subpopulation of CeA_L_ neurons that exert tonic inhibitory influence on the CeA_M_. Indeed, a population of CeA_L_ interneurons which exert inhibitory influence on the CeA_L_ neurons that inhibit CeA_M_ neurons has been identified (Ciocchi et al., 2010; Haubensak et al., 2010). It is noteworthy that the pPVT provides a dense and selective projection to the CeA_L_ (Li and Kirouac, 2008) and that this projection uses excitatory amino acids as neurotransmitters (Christie et al., 1987; Frassoni et al., 1997). Consequently, it is possible that the pPVT enhances fear by the activation of CeA_L_ GABA interneurons which inhibit CeA_L_ GABA neurons that are tonically inhibiting the CeA_M_ (disinhibition of the CeA_M_).

It is also important to mention that a previous study demonstrated that inactivation of the dorsal midline thalamus a few hours after fear conditioning had no effect of subsequent fear expression (Padilla-Coreano et al., 2012). Consequently, it is possible that the fear suppressive effects of the pPVT lesions observed in the present study were due to interference of fear memory retrieval. While the design of the present study cannot provide a direct answer to this question, it is clear from the results of the fear acquisition experiment that the pPVT is not necessary for the expression of a newly acquired fear. As such, it is also plausible that the attenuating effects of pPVT lesions observed in this study may have been due to the impairment in fear memory retrieval and not fear expression *per se*. It is also possible that fear expression to a newly conditioned cue does not involve the pPVT but that the influence of the pPVT may increase as the memory becomes consolidated at a systems level.

Previous studies using cFos as an anatomical marker of excitation have reported that the PVT becomes activated in rats that experience footshocks or when placed in the context in which footshocks were given (Beck and Fibiger, 1995; Yasoshima et al., 2007). Of the dorsal midline thalamic nuclei, only the PVT appears to be activated by the recall of the shock experience (Yasoshima et al., 2007). The pathway by which the recall of an aversive event like footshocks leads to the activation of the pPVT is speculative. Our laboratory has recently reported that the pPVT receives a strong projection from the prelimbic cortex (Li and Kirouac, 2012) while other groups have reported that this area of the prefrontal cortex is involved in the expression of learned fear (Quirk et al., 2000; Milad and Quirk, 2002; Vidal-Gonzalez et al., 2006; Corcoran and Quirk, 2007; Burgos-Robles et al., 2009; Sierra-Mercado et al., 2011). As such, it is possible that the recall of fear related memories by neurons in the prelimbic cortex leads to activation of neurons in the pPVT. The view that the pPVT plays a role in fear expression is consistent with previous studies indicating that this area of the thalamus also exerts an influence on other negative emotional behaviors including anxiety (Li et al., 2009, 2010a,b; Heydendael et al., 2011) and conditioned place avoidance to morphine withdrawal (Li et al., 2011). The fact that the pPVT is strongly connected to areas of the brain that mediate fear, anxiety and avoidance behaviors points to a specific role for the pPVT in the regulation of negative emotions (Li and Kirouac, 2008). Consistent with this view, we found that lesions of the pPVT did not affect the motivation of rats to bar-press for food (positive emotional behavior).

## Conflict of interest statement

The authors declare that the research was conducted in the absence of any commercial or financial relationships that could be construed as a potential conflict of interest.
